# MicroRNAs in Atrial Fibrillation: Mechanisms, Vascular Implications, and Therapeutic Potential

**DOI:** 10.3390/biomedicines12040811

**Published:** 2024-04-06

**Authors:** Emmanouil P. Vardas, Panagiotis Theofilis, Evangelos Oikonomou, Panos E. Vardas, Dimitris Tousoulis

**Affiliations:** 1 1st Cardiology Department, General Hospital of Athens “Hippokration”, University of Athens Medical School, 11528 Athens, Greece; vardas.man@gmail.com (E.P.V.); panos.theofilis@hotmail.com (P.T.); 2 Department of Cardiology, General Hospital of Athens “G. Gennimatas”, 11527 Athens, Greece; 3 3rd Cardiology Department, Sotiria Regional Hospital for Chest Diseases, University of Athens Medical School, 11527 Athens, Greece; boikono@gmail.com; 4 Biomedical Research Foundation Academy of Athens, Heart Sector, Hygeia Hospitals Group, Attica, 15123 Athens, Greece; pvardas@hygeia.gr

**Keywords:** atrial fibrillation, fibrosis, inflammation, oxidative stress, microRNA

## Abstract

Atrial fibrillation (AFib), the most prevalent arrhythmia in clinical practice, presents a growing global health concern, particularly with the aging population, as it is associated with devastating complications and an impaired quality of life. Its pathophysiology is multifactorial, including the pathways of fibrosis, inflammation, and oxidative stress. MicroRNAs (miRNAs), small non-coding RNA molecules, have emerged as substantial contributors in AFib pathophysiology, by affecting those pathways. In this review, we explore the intricate relationship between miRNAs and the aforementioned aspects of AFib, shedding light on the molecular pathways as well as the potential diagnostic applications. Recent evidence also suggests a possible role of miRNA therapeutics in maintenance of sinus rhythm via the antagonism of miR-1 and miR-328, or the pharmacological upregulation of miR-27b and miR-223-3p. Unraveling the crosstalk between specific miRNA profiles and genetic predispositions may pave the way for personalized therapeutic approaches, setting the tone for precision medicine in atrial fibrillation.

## 1. Introduction

Atrial fibrillation (AFib), the most common cardiac arrhythmia, represents a significant medical concern, affecting an ever-growing number of individuals worldwide. With the demographic shift towards an older global population, the prevalence of AFib is expected to rise exponentially, presenting a significant challenge to healthcare systems and professionals [1]. The multifaceted pathophysiology of AFib reflects the complex interactions of cellular and molecular mechanisms that underlie its onset and progression.

Among the numerous factors that contribute to AFib’s complexity, microRNAs (miRNAs) have gained recognition in the scientific community. These small, non-coding RNA molecules are vital in regulating gene expression, influencing multiple biological processes that are fundamental to AFib establishment. MiRNAs have demonstrated their strength as multifunctional agents by having a major impact on vascular physiology and fibrotic tissue changes, and regulating inflammatory responses and oxidative stress pathways. This detailed review examines the complex relationship between miRNAs and the aforementioned aspects of AFib. By exploring the molecular details, we highlight the significant potential of miRNAs, both as diagnostic tools and as transformative therapeutic targets that could reshape the future of AFib management.

## 2. MicroRNA-Mediated Fibrotic Mechanisms in Atrial Fibrillation

Fibrosis is an aberrant biological process that manifests through the disproportionate buildup of extracellular matrix (ECM) constituents, especially collagen, within the afflicted tissue. In the cardiac region, fibrosis may result from an array of inciting factors, including but not limited to inflammation, oxidative stress, ischemia, and aging. The development of fibrosis is contingent on the activation of fibroblasts, the principal cell type responsible for synthesizing ECM components within the heart, and the subsequent deposition of these components in the interstitial space between cardiomyocytes [2].

Concerning Afib, fibrosis can play a role in the onset and persistence of an arrhythmia by modifying the electrical and mechanical traits of the atrial tissue. In particular, fibrosis can impact the following mechanisms:

1. Disruption of atrial conduction: Fibrosis can modify the electrical and mechanical properties of the atrial tissue, contributing to the initiation and maintenance of the arrhythmia. One of the key mechanisms through which fibrosis exerts its effect is by disrupting atrial conduction. Fibrosis can create regions of slowed conduction, a conduction block, and conduction heterogeneity in the atrial tissue, which promotes the formation of re-entrant circuits that sustain AFib. The underlying mechanism for this effect is thought to be related to the increased interstitial resistance and reduced intercellular coupling caused by fibrosis. In fibrotic tissue, the gap junctions that allow for rapid cell-to-cell communication become disrupted, leading to a decrease in the velocity and uniformity of the electrical signal propagation. This, in turn, can create areas of slow or blocked conduction that serve as the substrate for the formation of re-entrant circuits, which sustain AFib [3].
2. Enhancement of automaticity: Fibrosis can facilitate the emergence of ectopic foci in the atrial tissue by modifying the electrophysiological characteristics of the cardiomyocytes. In particular, fibrosis can induce alterations in the ion channels and calcium handling proteins that govern the generation and propagation of action potentials in the atrial myocytes. These modifications can enhance the automaticity of the cardiomyocytes, rendering them more susceptible to generating spontaneous impulses that give rise to ectopic foci capable of triggering Afib [4].
3. Alteration of repolarization: Fibrosis can modify the repolarization properties of the atrial myocytes, resulting in an elongation of the action potential duration and a heterogeneity of repolarization across the atrial tissue. These changes can produce a substrate that promotes the development of arrhythmias by facilitating the formation of re-entrant circuits and ectopic foci. Moreover, fibrosis can increase the susceptibility of the atrial tissue to the effects of triggers, such as adrenergic stimulation or rapid pacing, by altering the electrophysiological properties of the cells. Consequently, these triggers can more readily cause disruptions in the conduction of electrical impulses across the atrial tissue, potentially leading to the onset or perpetuation of Afib [5].
4. Mechanical stiffness and stretch-induced remodeling: Fibrosis can modify the mechanical properties of the atrial tissue, reducing its compliance and increasing its susceptibility to stretch-induced remodeling. This can predispose the tissue to atrial dilatation and hypertension, both of which are established risk factors for atrial fibrillation. Additionally, fibrosis can instigate a pro-inflammatory and pro-fibrotic milieu within the atrial tissue, amplifying the fibrotic process and fueling the development of Afib [6].

**Figure 1:**
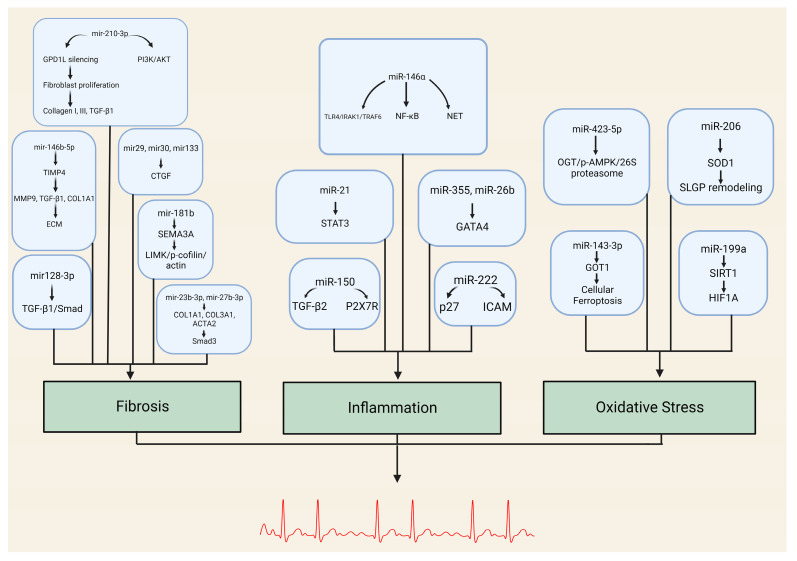
Illustration that demonstrates the complex relationships between the expression of particular microRNAs, signaling pathways, transcription factors, and proteins that collaboratively promote fibrosis, oxidative stress, and inflammation. The dynamics of these molecules promote the development of atrial fibrillation. GPD1L: Glycerol-3-phosphate dehydrogenase 1 like, TGF-β1: Transforming growth factor beta 1, PI3K: Phosphoinositide 3-kinase, Akt: Protein kinase B, TIMP4: Tissue inhibitor of metalloproteinase 4, MMP9: Matrix metallopeptidase 9, COL1A1: Collagen type I alpha 1 chain, ECM: Extracellular matrix, SMAD: Suppressor of mothers against decapentaplegic, CTGF: Connective tissue growth factor, SEMA3A: Semaphorin-3A, LIMK: LIM kinase, p-cofilin: Phospho-cofilin, COL3A1: Collagen type III alpha 1 chain, ACTA2: Alpha (2)-smooth muscle actin, TLR4: Toll-like receptor 4, IRAK1: Interleukin-1 receptor-associated kinase 1, TRAF6: Tumor necrosis factor receptor associated factor 6, NF-kB: Nuclear factor kappa B, NET: Neutrophil extracellular trap, STAT3: Signal transducer and activator of transcription 3, GATA4: GATA binding protein 4, P2X7R: Purinergic P2X7 receptor, P27: Protein 27, ICAM: Intercellular adhesion molecule, TGF-β2: Transforming growth factor-beta 2, OGT: O-GlcNAc transferase, p-AMPK: Phosphorylated AMP-activated protein kinase, SOD1: Superoxide dismutase type 1, SLGP: Superior left ganglionated plexus, GOT1: Glutamic-oxaloacetic transaminase 1, SIRT1: Sirtuin 1, HIF1A: Hypoxia inducible factor 1 subunit alpha.

The molecular mechanisms underlying fibrosis in AFib are not fully understood, but emerging evidence suggests that miRNAs play a critical role in this process (Table 1, Figure 1). The role of miRNA-146b-5p in promoting atrial fibrosis has been elucidated in recent studies. This miRNA represses a tissue inhibitor of matrix metalloproteinase 4 (TIMP4), leading to an upregulation of fibrotic markers such as matrix metalloproteinase-9 (MMP9), transforming growth factor beta-1 (TGFB1), and alpha-1 type I collagen (COL1A1). The consequential overexpression of miR-146b-5p results in collagen fiber accumulation in the extracellular space, intensifying cardiac fibrosis. Notably, inhibiting miR-146b-5p can attenuate cardiac fibrosis post-myocardial infarction, suggesting its potential as a therapeutic target for atrial fibrosis and AFib [7]. Further research demonstrates the significance of miR-210-3p in Afib. Through silencing of Glycerol-3-Phosphate Dehydrogenase 1 Like (GPD1L), miR-210-3p regulates atrial fibroblast proliferation and activation, and collagen synthesis. The overexpression of this miRNA results in the upregulation of collagen I, collagen III, and TGFB1. Additionally, its role in AFib is intricately linked to the Phosphoinositide 3-kinase (PI3K)/AKT signaling pathway, with its overexpression decreasing PI3K and AKT phosphorylation levels, thereby promoting fibrosis and AFib [8].

Stanley Nattel offers a comprehensive discussion on the role of various miRNAs in cardiac remodeling, especially in fibrotic responses linked to AFib. He emphasizes that most fibrosis-regulating miRNAs, including miR-30, miR-133, and miR-29, bind directly to profibrotic targets, enhancing fibrosis-promoting pathways. Nattel also highlights the unique role of miR-21, which is upregulated in AFib-promoting conditions and targets genes that lead to fibrosis [9]. Additionally, recent findings by Ying-Ju Lai et al. revealed that miR-181b induces atrial fibrosis by disrupting semaphorin 3A (Sema3A). Elevated miR-181b levels, upregulated by TGFB, lead to the downregulation of Sema3A, disrupting the Sema3A/LIM Domain Kinase (LIMK)/p-cofilin/actin axis and promoting atrial fibrosis [10].

The intricate relationship between miR-128-3p and plasmacytoma variant translocation 1 (PVT1) in Afib has also been explored. PVT1 acts as a molecular sponge for miR-128-3p, reducing its availability and preventing its inhibitory effect on specificity protein 1 (Sp1), subsequently activating the TGFB1/Smad signaling pathway, which is crucial in fibrosis [11].

Lastly, research conducted by Zhenzhen Yang et al. suggests that miR-23b-3p and miR-27b-3p play a pivotal role in promoting atrial fibrosis development, thereby stabilizing the re-entrant circuits that sustain Afib. These miRNAs promote atrial fibrosis by enhancing the expression of fibrosis-related genes, including COL1A1, COL3A1, and Actin Alpha 2 (ACTA2), in human atrial fibroblasts (HAFs). Through a positive feedback loop, Smad3 activation modulates the expression of miR-23b-3p and miR-27b-3p. These microRNAs, in turn, stimulate the Smad3 signaling by targeting TGFBR3 in HAFs [12].

The intricate interplay between miRNAs and atrial fibrosis offers promising avenues for therapeutic interventions in Afib. As our understanding of these molecular mechanisms deepens, targeted therapies can be developed to mitigate the progression of atrial fibrosis and AFib development.

## 3. MicroRNAs in Atrial Fibrillation’s Inflammatory Pathways

Inflammation is implicated in multiple stages of AFib’s pathophysiology, including initiation, maintenance, and thromboembolic complications. Evidence suggests that inflammation, both systemic and localized, can induce structural and electrical remodeling of the atrial myocardium, a critical mechanism contributing to AFib [13].

Systemic inflammation can trigger AFib and contribute to its persistence. Elevated levels of inflammatory markers, such as C reactive protein (CRP), inteurleukin-6 (IL-6), and tumor necrosis factor-alpha (TNF-α), are associated with a heightened risk of AFib [14]. Furthermore, systemic inflammatory conditions like autoimmune diseases and infections have been linked to AFib, suggesting that systemic inflammation disrupts normal atrial physiology [15].

In terms of localized inflammation, infiltrating inflammatory cells in the atrial tissue release cytokines and reactive oxygen species, inducing fibrosis and apoptosis of atrial myocytes. These changes disturb the normal electrical and structural properties of the atrium, contributing to the formation of a pro-arrhythmic substrate for AFib [16]. Furthermore, this inflammatory milieu triggers ion channel dysfunction and promotes the development of ectopic foci, which are critical for the initiation and maintenance of AFib [17].

Inflammation also enhances the thromboembolic risk in Afib. Inflammatory processes can trigger platelet activation and a hypercoagulable state, thereby promoting thrombus formation, a severe complication of AFib. Inflammatory biomarkers are being studied for their potential role in assessing stroke risk in patients with AFib, adding a new dimension to the current risk stratification schemas [18].

Given this role of inflammation in AFib, anti-inflammatory therapies have emerged as potential therapeutic strategies. The use of statins, known for their anti-inflammatory properties, has been associated with a lower incidence of AFib [19]. However, these findings need validation in large-scale clinical trials.

Although our understanding of the relationship between inflammation and AFib has improved significantly, challenges persist. Unraveling whether inflammation is a cause or a consequence of AFib, or both, is a topic of ongoing research. Moreover, the specific inflammatory pathways contributing to AFib remain to be fully elucidated, and inhibiting these pathways might have unintended side effects. MiRNAs have emerged as critical regulators in numerous biological processes, including inflammation. As our understanding of the pathogenesis of Afib continues to evolve, the contribution of miRNAs to the inflammation-mediated development and progression of AFib has become a focal point of recent research (Table 2, Figure 1).

MiR-146a, for instance, acts as a negative regulator in innate and inflammatory immune responses, primarily through the Nuclear factor kappa B (NF-κB) pathway. Pro-inflammatory elements, such as toll-like receptor-4 (TLR4), interleukin-1 receptor-associated kinase 1 (IRAK1), and Tumor necrosis factor receptor associated factor 6 (TRAF6), are direct targets of miR-146a. Arroyo et al. have suggested that miR-146a propels Neutrophil extracellular traps (NETs) in AFib, a process known to foster inflammation and thrombosis. This positions miR-146a as a potential regulator of the inflammatory processes that instigate AFib [20].

Similarly, miR-21 has been identified as a promoter of inflammation-associated atrial fibrosis. This promotion is achieved through the phosphorylation of the transcription factor Signal transducer and activator of transcription 3 (STAT3). It has been demonstrated that inhibiting miR-21 in rats with pericarditis and AFib led to a suppression of STAT3 phosphorylation, reduced expression of fibrosis-related genes, and decreased AFib vulnerability. There is also evidence suggesting a feedback loop, where the expression of miR-21 is positively regulated by phosphorylated STAT3. This intricate pathway potentially bridges atrial inflammation to fibrosis formation [21].

Further studies have highlighted the roles of miR-355 and miR-26b in the miRNA–inflammation network. Hao Zhang et al. used data from the Gene Expression Omnibus (GEO) database and miRNA–target relationships from the miRTarBase database to construct three miRNA regulatory networks to explore the inflammatory mechanism in the development of AFib. MiR-355 and miR-26b emerged as hubs in the miRNA–inflammation network, indicating their potential significance in the development of inflammation in AFib. MiR-26b modulates GATA Binding Protein 4 (GATA4) expression through a post-transcriptional mechanism, which is essential for the growth and survival of myocytes during cardiac hypertrophy. The influence of inflammation in AFib is further emphasized by its capacity to alter calcium homeostasis and connexins, both vital for atrial conduction; however, further research may be needed to determine the specific roles of miR-355 and miR-26b in AFib [22].

Another miRNA of interest is miR-150, which has been significantly dysregulated in cardiovascular conditions like unstable angina and post-myocardial infarction myocardial remodeling. Its marked downregulation in intermediate monocytes suggests a role in mediating inflammatory processes. Moreover, miR-150 has been delineated to encode for specific protein genes implicated in the etiology of AFib. Among the 18 identified genes, 11 have a regulatory function in the inflammatory signaling pathway. These proteins are instrumental in initiating the inflammatory cascade, with several being associated with apoptotic mechanisms and others with fibrotic pathways. Similar to miRNA-21, miRNA-150 is related to changes in levels of interleukins, such as TGF-β, which are involved mainly in the fibrotic processes. Therefore, miRNA-150 may promote atrial fibrillation through its regulation of the inflammatory response system and its impact on fibrosis [23,24].

Lastly, miR-222 has been linked with the development of degenerative valvular heart disease (DVHD) and has an association with AFib. A study conducted by Hualan Zhou et al. demonstrates the role of miR-222 in inflammation-mediated vascular remodeling. Elevated serum levels of miR-222 have been observed in patients with degenerative valvular heart disease (DVHD) and AFib compared to those without AFib. This elevation correlates positively with markers like IL-6, hs-CRP, and the N-terminal prohormone of brain natriuretic peptide (NT-proBNP), suggesting a role for miR-222 in exacerbating DVHD with AFib through inflammatory responses and vascular remodeling [25].

The intricate web of miRNAs and their association with inflammatory processes in AFib offers promising avenues for therapeutic interventions and a deeper understanding of AFib’s inflammatory mechanisms.

## 4. Oxidative Stress and MicroRNA Interplay in Atrial Fibrillation

In recent studies, the impact of oxidative stress on the initiation and development of Afib has been a focal point. Reactive oxygen species (ROS), which emerge as by-products during oxygen metabolism, have a pronounced effect on cardiomyocytes. The intricate molecular pathways through which elevated ROS levels contribute to AFib have been a subject of thorough research. Evidence indicates that specific ROS, such as hydrogen peroxide, have the capability to enhance the late Na^+^ current, leading to both early (EADs) and delayed afterdepolarizations (DADs). Concurrently, ROS can downregulate the overall Na^+^ current, potentially leading to the formation of re-entry circuits. In addition to these effects, ROS can amplify the L-type Ca^2+^ current and disrupt the intracellular calcium balance, which can result in EADs [26].

A major source of ROS production in AFib is Nicotinamide adenine dinucleotide phosphate oxidase (NOX). ROS derivatives from NOX, such as superoxide and hydrogen peroxide, initiate several processes encompassing atrial inflammation, fibrosis, and both structural and electrical remodeling. Upstream activators like angiotensin II (Ang II) and atrial stretch further promote NOX activation, creating a feedback loop where NOX activation promotes AFib, and vice versa. ROS also trigger several proinflammatory signaling pathways, including NF-κB and mitogen-activated protein kinase (MAPK) pathways, intensifying atrial remodeling and inflammation [27]. Additionally, ROS can activate MMPs, which degrade the extracellular matrix, fostering fibrosis. The damaging effects of ROS extend to proteins, lipids, and DNA, leading to cellular dysfunction and apoptosis. For instance, ROS-induced mitochondrial DNA damage in AFib can cause calcium overload by altering calcium handling proteins or channels, furthering atrial remodeling [28].

Delving deeper into the molecular mechanisms, Wenjun Xie et al. highlight the role of Ryanodine receptor 2 (RyR2), a calcium release channel in the sarcoplasmic reticulum (SR) of atrial myocytes. ROS oxidize RyR2, increasing the channel’s sensitivity to calcium, leading to an intracellular Ca^2+^ leak from the SR. This leak subsequently activates the Na^+^/Ca^2+^ exchanger (NCX), causing the depolarization of the atrial myocyte membrane. This results in the emergence of EADs and DADs, which can trigger AFib [29].

Moreover, the multifunctional Ca^2+^/Calmodulin-Dependent Protein Kinase II (CaMKII) is influenced by elevated ROS levels. ROS oxidize specific methionines in CaMKII’s regulatory domain, converting it into a continuously active state, termed ox-CaMKII. This activated state allows ox-CaMKII to phosphorylate serine 2814 on the RyR2, leading to increased diastolic SR Ca^2+^ release. This surge in calcium release can induce DADs, facilitating the emergence of Afib [30].

While oxidative stress stands as a significant facilitator of Afib, its underlying mechanisms are multifaceted and complex. One intriguing aspect of this intricate interplay involves miRNAs. Specifically, MiR-206 has demonstrated regulatory control over ROS levels by modulating Superoxide Dismutase 1 (SOD1) in primary canine myocardial cells. SOD1 plays a pivotal role in mitigating cellular ROS, and any dysregulation of miR-206 within local tissues may lead to heightened ROS production through the downregulation of the antioxidant SOD1 protein. This, in turn, can potentially induce neural remodeling in the Superior Left Ganglionated Plexuses (SLGPs). The process of nerve remodeling within GPs, particularly in SLGPs, appears to be a more significant contributor to the initiation and perpetuation of AFib compared to other atrial regions. Hence, it is plausible that the upregulation of miR-206, along with the subsequent enhancement in ROS generation, could contribute to the development and persistence of AFib by instigating Cardiac Autonomic Nerve Remodeling (ANR) specifically within SLGPs [31].

Additionally, Peng Luo and Wei Zhang have elucidated in their research that miR-423-5p regulates hydrogen peroxide-induced apoptosis in cardiomyocytes through precise targeting of O-GlcNAc transferase (OGT) and its downstream effectors, including phosphorylated adenosine monophosphate-activated protein kinase (p-AMPK) and the 26S proteasome. The downregulation of OGT by miR-423-5p activates AMPK and simultaneously inhibits the function of the 26S proteasome, ultimately leading to cardiomyocyte apoptosis, a process that is involved in the development of Afib [32].

Yuan Song and colleagues conducted a study revealing the regulatory role of miR-143-3p in oxidative stress, cellular ferroptosis, and the progression of Afib. Their findings indicate that miR-143-3p is underexpressed in cardiomyocytes afflicted with Afib, contrasting with its expression in normal cardiomyocytes. This suggests that the expression profile of miR-143-3p is influenced by Afib. Furthermore, an intriguing observation emerged from their research, wherein the overexpression of miR-143-3p demonstrated a capacity to mitigate oxidative-stress-induced cell ferroptosis in Afib-afflicted cardiomyocytes. The proposed mechanistic basis for this phenomenon lies in the upregulation of miR-143-3p, which, in turn, leads to the degradation of glutamic-oxaloacetic transaminase 1, an enzyme involved in cardiomyocyte ferroptosis [33].

Lastly, MiR-199a has been identified as a regulator of sirtuin 1 (SIRT1) gene expression. In cardiomyocytes, miR-199a downregulates the expression of SIRT1, leading to a decrease in its protein levels. This downregulation of SIRT1 by miR-199a has been associated with increased apoptosis and oxidative stress in cardiac cells. In the context of AFib, a decreased expression of miR-199a has been observed in tissue samples from patients with postoperative AFib after coronary artery bypass graft surgery. This decrease in miR-199a expression is accompanied by an increase in SIRT1 protein and hypoxia-inducible factor-1alpha levels, which can lead to hypoxia preconditioning in cardiac myocytes [34].

## 5. MicroRNAs and Their Vascular Implications in Atrial Fibrillation

MiRNAs are also known to have a notable impact on the vasculature in Afib. These miRNAs contribute to the development and progression of AFib by influencing specific genes involved in endothelial cell function, vascular smooth muscle cell proliferation, angiogenesis, vascular inflammation, and vascular remodeling.

MiRNAs regulate endothelial cell function in AFib by targeting genes that control processes like endothelial cell proliferation, migration, and angiogenesis. For instance, miR-126 plays a pivotal role in vascular biology, particularly in the context of angiogenic signaling and maintaining vascular integrity by modulating the expression of vascular endothelial growth factor (VEGF) and its corresponding receptor, VEGFR2, within endothelial cells. Furthermore, miR-126 influences the endothelial expression of vascular cell adhesion molecule 1 (VCAM-1), a key molecule in leukocyte recruitment to inflammatory sites. This miRNA also influences the interplay between hemodynamics and VEGF signaling during the angiogenic process. Dysregulated serum levels of miR-126 may lead to aberrant angiogenesis, potentially elevating the risk of AFib. Moreover, a knockdown of miR-126 can compromise endothelial cell migration, impacting vessel growth, development, and organization—factors intrinsically linked to AFib pathogenesis [35].

MiRNAs also impact vascular smooth muscle cell (VSMC) proliferation in AFib, which is crucial in the remodeling of blood vessels observed in this condition. MiR-221 and miR-222 have been identified as pivotal regulators in VSMC growth and neointimal hyperplasia. In a study conducted by Xiaojun Liu et al., it was observed that the expression of these miRNAs in cultured VSMCs was elevated by growth stimulators. Furthermore, when miR-221 and miR-222 were underexpressed, there was a noticeable decrease in VSMC proliferation in vitro. This growth regulation was linked to their targeting of genes p27 (Kip1) and p57 (Kip2) [36].

Additionally, under hypoxic conditions, miR-210 is upregulated in cardiomyocytes, serving as a cellular adaptation to low oxygen levels. Molecularly, miR-210 enhances angiogenesis by upregulating key angiogenic factors. Concurrently, it protects against apoptosis by inhibiting caspase activity. Two primary molecular targets of miR-210, Ephrin A3 (Efna3) and Protein tyrosine phosphatase 1B (Ptp1b), further elucidate its role. Efna3 modulates angiogenic processes, while Ptp1b is involved in apoptosis pathways. Collectively, miR-210 acts as a regulator, promoting angiogenesis and cell survival, making it pivotal in Afib research [37].

Moreover, vascular inflammation is closely associated with AFib, and miRNAs contribute to this process by regulating the expression of genes involved in immune cell recruitment and activation within the vasculature. MiR-155, miR-146a, and miR-21, among others, are miRNAs implicated in vascular inflammation in AFib by targeting inflammatory cytokines, adhesion molecules, and TLRs as previously reported [38,39].

In summary, miRNAs play a significant role in the vascular effects observed in atrial fibrillation. They affect endothelial cell function, vascular smooth muscle cell proliferation, angiogenesis, vascular inflammation, and vascular remodeling. Understanding the dysregulation of miRNAs in this context may provide insights into the underlying mechanisms of AFib and potential therapeutic targets for intervention.

## 6. MicroRNAs as Emerging Biomarkers and Therapeutic Facilitators of Atrial Fibrillation; Future Perspectives

Intense scientific research over the past two decades has focused on the regulatory role of mRNAs and has undoubtedly revealed a plethora of new findings. Providing optimism, these small, non-coding RNA molecules that regulate post-transcription gene expression could potentially emerge as valuable biomarkers and therapeutic facilitators in the management of atrial fibrillation.

As previously mentioned, the pathophysiology of AFib involves complex mechanisms with intricate pathways, including inflammatory processes leading to atrial fibrosis, structural and electrical remodeling, and intracellular calcium handling abnormalities. In the previous pathophysiological pathways, the role of a number of mRNAs is recognizable and well documented. However, to date, the existing relevant literature, although rich in exploitable findings, is characterized by a plethora of inconsistent results, which unfortunately limit the clarity of conclusions. In light of the previous findings, there is now a general consensus on the need for well-standardized research protocols’ development, sample processing, and analysis of mRNA levels in biofluids to increase the reliability of relevant research and, primarily, the reproducibility of findings.

### 6.1. miRNAs as Potential Biomarkers of Atrial Fibrillation

Translating the interesting research findings regarding mRNAs and their significance in the development of atrial structural and electrical remodeling into clinically relevant data is far from reality. Clearly, at present, there are no suitable biomarkers that could be well utilized in advance for the timely prognosis and primary diagnosis of AFib. However, there are exploitable findings indicating that these small RNA molecules possess high stability, sensitivity, and specificity, making them attractive biomarkers for AFib prognoses, primary diagnoses, and post-ablation monitoring.

It is noteworthy that the reliability of all the previous findings requires well-organized and well-standardized multicentric studies. Currently, we could utilize the plethora of research findings and refer to the most confirmed cases of miRNAs that multiple studies have identified as significant factors in the pathophysiology of AFib. For example, the review article by Medeiros da Gomez da Silva highlighted three miRNAs (miR-21, miR-150, miR-328) with altered patterns of expression in more than one study of patients who potentially developed atrial fibrillation, suggesting the possible reliable role of these three miRNAs as biomarkers of AFib pathophysiology [40].

Specifically focusing on the significance of miR-21 as a reliable AFib biomarker, it should be noted that a significant number of research studies consistently recognize its importance. The relevant findings suggest that miRNA-21 seems to be upregulated in the atrial tissue of patients with AF, particularly those with persistent AFib, as well as in those with atrial fibrosis. Summarizing the findings of the existing literature, it is reasonable to support that through a significant number of miRNAs identified to be involved in the mechanisms of AFib development, miRNA-21 is the most recognizable as a critical biomarker of clinical significance. However, the previous promising finding still faces a number of challenges that need to be addressed before it could be widely used in clinical practice.

### 6.2. miRNAs as Biomarkers of the Risk of Major Adverse Thrombotic Cardiovascular Events in Atrial Fibrillation

There are few biomarkers able to forecast new thrombotic events and MACE in patients with AFib, although the relevant literature is rather limited and recent, with findings from various studies lacking significant mutual confirmation. For instance, Rivera-Caravaca J et al. conducted a pilot study examining 179 miRNAs in the plasma of 9 patients with AFib and MACE compared to 10 patients with AFib without similar events and followed them for 8 years [41]. According to the study’s findings, patients with the highest risk of MACE had significantly higher levels of miR-22-3p and miR-107 and lower levels of miR-146a-5p. Furthermore, the inclusion of miR-107 and miR-146a-5p levels in the 2MACE score significantly improved the predictability of MACE compared to the original score.

Additionally, Roldan V et al. conducted a notable study aimed at assessing the prognostic role and biological effect of functional miR-146a polymorphisms, rs2431697 and rs2910164, in patients with non-valvular AFib receiving oral anticoagulation [42]. The results of this study established the aforementioned miR-146a polymorphisms as a prognostic biomarker for acute major cardiovascular events in patients under oral anticoagulation.

Based on the previous studies and a number of other relevant studies, the emergence of miRNAs as important agents in the pathophysiology of MACE development in patients with AFib with or without anticoagulation treatment becomes apparent. All these findings, as repeatedly mentioned in this review, need to be confirmed through well-standardized multicenter trials.

### 6.3. miRNAs as Potential Therapeutic Agents in Atrial Fibrillation Management

**Figure 2:**
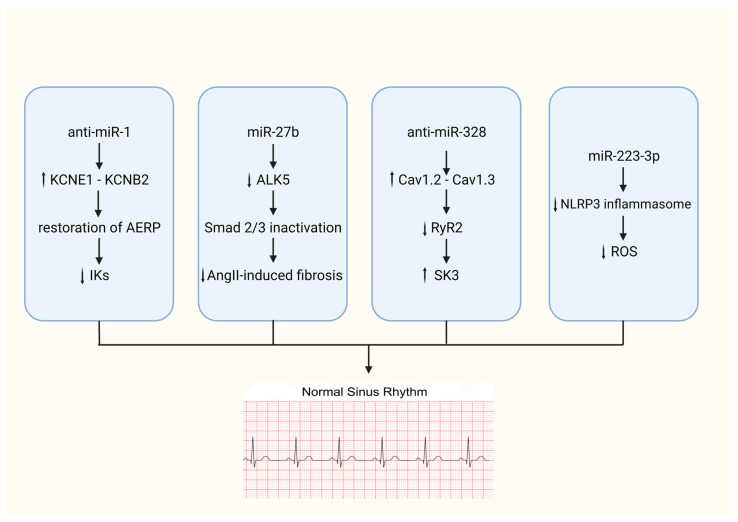
Illustration depicting the coordinated molecular pathways involving specific miRNA and anti-miRNA molecules. The cascades that are shown demonstrate the regulatory impact on important cellular functions that eventually result in the restoration of sinus rhythm. KCNE1: Potassium voltage-gated channel subfamily E member 1, KCNB2: Potassium voltage-gated channel subfamily B member 2, AERP: Atrial effective refractory period, IK: Intermediate conductance potassium channel, SMAD: Suppressor of mothers against decapentaplegic, ALK5: Transforming growth factor beta receptor 1, AngII: Angiotensin II, Cav1.2: Calcium channel, voltage-dependent, L type, alpha 1C subunit, Cav1.3: Calcium channel, voltage-dependent, L type, alpha 1D subunit, RyR2: Ryanodine receptor 2, SK3: Small conductance calcium-activated potassium channel 3, NLRP3: NOD-, LRR- and pyrin-domain-containing protein 3, ROS: Reactive oxygen species.

miRNAs hold great promise as therapeutic agents in AFib. Their ability to influence gene expression and cellular pathways central to AFib pathogenesis offers a unique opportunity for possible therapeutic interventions (Figure 2). Generally, the therapeutic concept of targeting miRNAs as a therapeutic tool could be achieved by either inhibiting overexpressed miRNA using antagonisms (anti-miRNA) or mimicking the function of downregulated miRNA through miRNA mimics.

To our knowledge, there is currently no clinically accepted therapy using the aforementioned methodologies that could suspend the development or maintenance of AFib. However, appropriate randomized trials are required for the acceptance of such therapy, studying safety issues, as well as the effectiveness of each treatment. It should be noted here that for each miRNA therapeutic intervention, it will be necessary to ensure in detail that the goal of the specific therapeutic factor is to modify exclusively a specific pathophysiological pathway and that the administered miRNA does not have the ability to modify other non-targeted pathways of human physiology.

Considering previous statements, it is interesting to refer to characteristic studies where the modification of the expression of certain miRNA in vivo studies has led to beneficial results. The case of miRNA-21 is of paramount importance, as it has been repeatedly recognized that its upregulation leads to atrial fibrosis and atrial fibrillation. The study by Pradhan et al. showed that the control of miR-21 using anti-miR-21 induced anti-fibrotic processes in vitro [43] while additionally Xiona Xu et al. [44] highlight the beneficial effect of anti-miR-21 in AFib reduction in animals. Additionally, several other animal studies have also led to encouraging results since the in vivo manipulation of miRNAs in experimental AFib can lead to a limitation of structural and electrical remodeling. Specifically, Luo et al. discovered that antagonism of -328 may successfully reverse AFib susceptibility in ATP dogs through the in vivo adenoviral-mediated forced expression of miR-328 [45]. Furthermore, Jia et al., by administering LNA-based antiviral-1, found how to prolong the atrial effective refractory period and reduce susceptibility and arrhythmia duration in rabbits [46].

The previous studies represent characteristic examples of a significant number of similar studies and highlight the importance of experimental research in this field and the tremendous clinical perspectives in this domain.

## 7. Conclusions

Recent investigations into the molecular mechanisms of AFib have highlighted the pivotal role of miRNAs in its pathophysiology. These small, non-coding RNA molecules are intricately involved in post-transcriptional regulation, influencing endothelial function, myocardial remodeling, and electrophysiological properties of the heart. Their ability to modulate gene expression has wide-ranging effects on different aspects of AFib, encompassing vascular dynamics, fibrosis in cardiac tissue, and the complex pathways of inflammation and cellular responses to oxidative stress. As our comprehension of molecular intricacies advances, the therapeutic potential of miRNAs in AFib becomes increasingly evident, indicating their usefulness in formulating innovative, precisely targeted interventions. Furthermore, progress in molecular biology is bringing us closer to the prospect of integrating miRNAs into routine treatment protocols. This could potentially revolutionize our approach to AFib, paving the way for more personalized and effective treatment strategies.

## Figures and Tables

**Table 1:** Key miRNAs Regulating Fibrotic Processes in Atrial Fibrillation.

miRNA	Target Genes/Pathways
miR-146b-5p	TIMP4, MMP9, TGFB1, COL1A1
miR-210-3p	GPD1L, PI3K/AKT pathway
miR-29, miR-30, miR-133, miR-21	Connective tissue growth factor (CTGF)
miR-23b-3p, miR-27b-3p	COL1A1, COL3A1, ACTA2 TGFBR3/Smad3 pathway
miR-128-3p	PVT1, Sp1 TGF-β1/Smad pathway
miR-26	CNJ2/IK TRPC3
miR-181b	Sema3A Sema3A/LIMK/p-cofilin/actin axis

**Table 2:** miRNAs Involved in Inflammation-Mediated Processes in Atrial Fibrillation.

miRNA	Target Genes/Pathways	Role in Inflammation
miR-146a	TLR4, IRAK1, TRAF6 NF-κB pathway	Negative regulator of innate inflammatory responses NET formation
miR-21	STAT3 pathway	Promotes inflammation-associated fibrosis
miR-355, miR-26b	GATA4	Alters fibroblast expression (cellular senescence) Affects atrial conduction through calcium homeostasis
miR-150	*TGF-β* *EGRR2*, *P2X7R*	Modulates inflammatory response system by targeting pro-inflammatory ATP receptor and multiple regulatory genes
miR-222	Associated with DVHD P27 ICAM-1	Role in inflammation-mediated vascular remodeling Downregulation of IRF-2
